# Psychological Well-Being and Dysfunctional Eating Styles as Key Moderators of Sustainable Eating Behaviors: Mind the Gap Between Intention and Action

**DOI:** 10.3390/nu17152391

**Published:** 2025-07-22

**Authors:** Elena Lo Dato, Sara Gostoli, Elena Tomba

**Affiliations:** Department of Psychology, University of Bologna, 40126 Bologna, Italy; elena.lodato2@unibo.it (E.L.D.); sara.gostoli2@unibo.it (S.G.)

**Keywords:** sustainable eating, theory of behavioral choice, psychological well-being, dysfunctional eating styles

## Abstract

**Background**: Promoting sustainable eating is gaining increasing attention, yet the transition from intentions to actual behaviors remains unclear. This study compares the theory of planned behavior (TPB) and the theory of behavioral choice (TBC) in predicting such intentions and examines the moderating role of distress, psychological well-being, and dysfunctional eating styles in the intention–behavior relationship. **Methods**: A total of 223 participants from the general population (29.49 ± 9.30 years; 68.6% females) completed an online survey assessing TPB and TBC predictors, the Sustainable and Healthy Dietary Behaviors (SHDB) questionnaire, the Depression and Anxiety Stress Scale (DASS-21), the Psychological Well-Being Scale (PWBS), and the Dutch Eating Behavior Questionnaire (DEBQ). **Results**: The TBC model explained a significantly greater variance in intention (R^2^ = 0.45, ΔR^2^ = 0.28, F(215,4) = 27.27, *p* < 0.001) compared to the TPB, with TBC-affect (β = 0.48, *p* < 0.001) and felt obligation (β = 0.23, *p* < 0.001) being the strongest predictors. Moderation analyses revealed that the intention–behavior link was stronger in participants with lower external eating and autonomy. **Conclusions**: Both internal and external factors play a crucial role in predicting intentions. In addition, the intention–behavior link is stronger in individuals who are less reactive to external food stimuli and more influenced by social pressure. Promoting more balanced psychological well-being and functional eating habits may foster more sustainable diets.

## 1. Introduction

Non-sustainable diets significantly contribute to climate change [1] and represent one of the leading causes of diet-related non-communicable diseases and obesity [2,3], which in turn place a substantial burden on healthcare systems worldwide [4]. The rising production of cheap, palatable, and energy-dense foods, which are made much more convenient and accessible by distribution systems and more appealing by food marketing, negatively impacts both environmental and individual well-being [2].

Despite awareness of the harm associated with non-sustainable diets, changing one’s eating habits can be challenging [5], and psychological research has been particularly interested in understanding the individual determinants that drive behavioral shifts towards more sustainable food choices [6,7,8,9]. According to a recent systematic review [10], the theory of planned behavior (TPB) [11] is one of the most frequently applied psychological frameworks. According to the TPB, desired behaviors are determined by underlying intentions, which in turn are predicted by personal attitudes (i.e., the degree to which a person has a favorable or unfavorable evaluation of that behavior), social norms (i.e., the perceived social pressure to perform that behavior), and perceived behavioral control (PBC, i.e., the perceived ease or difficulty in performing that specific behavior) [11].

However, this theory appears insufficient in explaining the gap between intention and action, especially in terms of sustainable eating behaviors. Extended versions of the TPB have been proposed and tested to expand its predictive power through the integration of additional psychological factors. The theory of behavioral choice (TBC) has recently been proposed and tested concerning pro-environmental food choices [12]. Building on the TPB-predictors of intention (personal attitudes, social norms, and PBC), the TBC integrates additional predictors of intention, including *attitudes plus* (i.e., personal values), *felt obligation* (i.e., the perceived obligation to perform a specific behavior), *affect* (i.e., the emotions associated with that behavior), and *habitual behaviors* (i.e., one’s habits) [12]. Including these additional predictors addresses key criticisms of the TPB [13]. For instance, the TPB’s assumption of purely rational choices overlooks the fact that many behaviors, including food choices, could be significantly influenced by automatic processes [14]. Integrating habitual behaviors may be valuable for predicting food choices in this context. The TPB has also been criticized for its limited focus on cognitive and affective processes in decision-making [15], a relevant gap for dietary changes [16]. Consequently, incorporating affect provides another potentially valuable predictor of intention. Lastly, the original TPB does not account for the role of moral influence in behavioral change, despite studies highlighting how moral obligation can increase the intention to engage in specific behaviors, including ecological food choices [17]. Thus, incorporating feelings of obligation can further enhance the model’s predictive power. Finally, the TBC suggests that the gap between intention and action can be attributed to psychological barriers, such as justifications and rationalizations [12].

While substantial efforts have been made to identify general psychological factors (i.e., emotions, mood, values) that may hinder the adoption of sustainable eating behaviors [6,8], limited attention has been paid to specific clinical psychological variables—particularly those related to mental health—that may also play a role in this domain. The bi-directional interplay between food choices, mental health, and psychological well-being has been widely highlighted [18]. Emotional states influence what we eat, while the nutritional properties of foods also impact our physical and mental health, potentially playing a role in several mental disorders [19]. Nonetheless, the influence of psychological moderators has not been extensively explored in the context of sustainable eating, despite numerous studies demonstrating their significant impact on healthy dietary practices. Psychological distress can lead to dysfunctional eating behaviors, such as the overconsumption of unhealthy foods and a lower adherence to healthy diets [20], with greater psychological distress shown to hinder healthy food choices both in healthy individuals and in patients with obesity [21,22]. Additionally, adherence to the Mediterranean diet, often identified as a sustainable dietary model [5,23], is inversely associated with psychological disorders, including depression, anxiety, and stress [24]. Therefore, psychological distress may play an essential role in determining the actualization of sustainable eating behaviors and may act as a potential moderator in the relation between intention and behavior. In contrast, lower levels of distress and higher levels of subjective well-being have been linked to greater adherence to healthy and sustainable dietary patterns among healthy individuals [25]. Furthermore, optimal levels of psychological well-being (PWB) have consistently been linked to a range of health-promoting behaviors [26], including healthy eating [25,26,27,28], while compromised PWB levels serve as barriers to healthy dietary behaviors [29,30,31]. In the context of sustainability, positive psychological consequences (i.e., happiness, psychological well-being) deriving from sustainable behaviors serve as significant determinants of pro-environmental behaviors [32], further supporting the potential moderating role of PWB in the actualization of sustainable eating behaviors.

In addition, dysfunctional eating styles—such as emotional, external, and restrained eating—may interfere with sustainable eating behaviors. These dysfunctional eating styles may lead to unhealthier food choices, thus potentially influencing sustainable food choices. The consumption of food in response to emotional cues rather than physiological hunger is often associated with preferences for energy-dense, high-fat, and high-sugar foods [33,34]. Similarly, eating in response to external stimuli (i.e., seeing other people eating) is associated with unhealthy food choices [35,36,37]. Conversely, the intentional restriction of food intake has shown associations with healthier dietary practices [38], but it also poses a risk for the onset of disordered eating symptoms, such as orthorexia nervosa [39].

**Figure 1:**
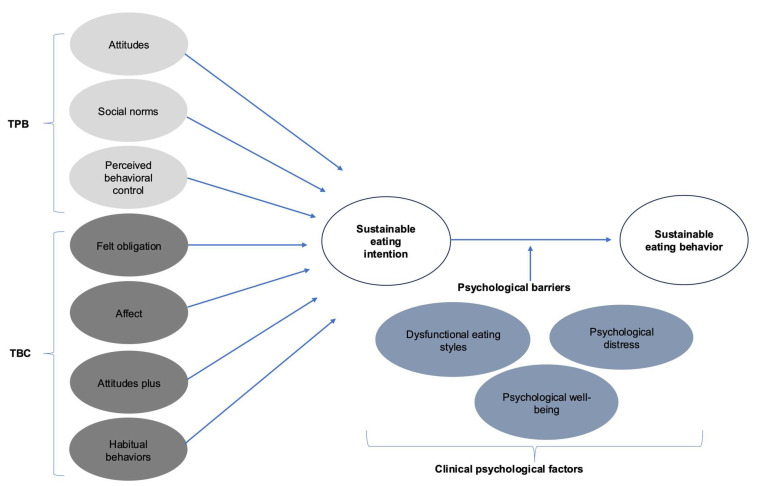
Predictive theoretical model of sustainable eating intentions and behaviors, integrating constructs from the TPB and the TBC, along with clinical psychological variables.

Considering the potential influence of these psychological factors, the current study proposes a predictive theoretical model of sustainable eating intentions and behaviors. This model integrates predictors of intention from the TPB and the TBC, while also incorporating dysfunctional eating styles, psychological distress, and psychological well-being as potential moderators in the relationship between intention and behavior (Figure 1).

## 2. Aims and Hypotheses

The aims and hypotheses of the present cross-sectional study were constructed before the development and implementation of the online survey. Specifically, the aims of the study were twofold: (1) comparing predictors of intention to engage in more sustainable eating behaviors as proposed by the TPB (attitudes, social norms, and PBC) and the TBC (felt obligation, affect, attitudes, and habitual behaviors) theoretical models; (2) testing the role of psychological distress, PWBS, and dysfunctional eating styles as possible moderators in the relation between intention and behavior in an Italian adult general population.

We hypothesize that: (1) TBC predictors of intention will fit the data better in predicting the intention to engage in more sustainable eating behaviors, and that (2) psychological distress, PWB, and dysfunctional eating styles will act as moderators in the relation between intention and behavior.

## 3. Methods

The study was conducted following the Helsinki Declaration (revised in Fortaleza, Brazil, in October 2013), and the relevant sections of the International Council for Harmonization—Good Clinical Practice (ICH-GCP) (Document EMA/CHMP/ICH/135/1995 of December 2016) were implemented during the study. Study participation was voluntary and could be cancelled any time without providing reasons and without negative consequences. The Bologna University Bioethics Committee approved the project on 27 June 2023 (protocol number 0177765). All participants gave their informed consent to participate in the study.

### 3.1. Participants

The study recruited participants from the general adult population in Italy online via QR codes leading to a Qualtrics page. The IP address of participants was not collected to ensure confidentiality and anonymity. The QR code was shared on major social media and social networks (i.e., Facebook, Instagram, and LinkedIn), and a snowballing approach was applied to researchers’ contacts. In addition, QR codes were shared among university students and their networks. Compensation was not offered for participation in this study. Interested participants were eligible to participate in the study if they: (1) were over 18 years old and (2) had a good command of the Italian language. Exclusion criteria were: (a) inability to give informed consent; (b) bad command of the Italian language; (c) age outside the required range.

A total of 431 participants started filling in the questionnaire. However, after excluding incomplete responses, 223 complete responses were included in the final dataset used for analyses, corresponding to a completion rate of 51.7%.

### 3.2. Measures

Participants filled in a 30 min online self-report questionnaire made available through Qualtrics. The questionnaire included an ad hoc form on sociodemographic, anthropometric (i.e., height and weight), and clinical (i.e., medical or mental diseases) data, and self-report psychometric measures for the assessment of predictors of intention, sustainable eating behaviors, psychological well-being, dysfunctional eating styles, and psychological distress.

Specifically, participants filled in the following questionnaires:

- *Predictors of Intention*. To assess predictors of intention to engage in more sustainable eating behaviors as proposed by TPB (attitudes, social norms, and perceived behavioral control) and TBC (attitude plus, affect, felt obligation, and habits) models, a 31-item ad hoc questionnaire on a 7-point Likert scale was used. The questionnaire was adapted from a previous study [12].
- *Sustainable and Healthy Dietary Behaviors* (SHDB) [40]. A 33-item questionnaire on a 6-point Likert scale consisting of five dimensions was used to assess sustainable and healthy eating behaviors. The five dimensions included food choices (e.g., choosing the right amount of food, choosing locally produced and organic food, avoiding processed food), storing (e.g., checking the expiration dates of food), cooking (e.g., minimizing the use of disposable materials, making a menu so that groceries will not be discarded), consumption (e.g., avoiding the use of plastic cutlery), and disposal (e.g., recycling garbage). Scores range between 1 and 5, with higher scores indicating greater sustainable eating behaviors. So far, the questionnaire has been validated only in the Japanese population, showing adequate internal consistency (Cronbach’s α = 0.92) [40]. The authors emphasized that providing information on healthy eating and sustainability, highlighting the associated benefits, is needed to promote behaviors such as choosing healthy foods and sustainable cooking practices. The questionnaire was translated into Italian using the back-translation method. This method involves an initial translation of the items from the original language into Italian by at least two researchers who must agree on the final version. This version is then reviewed by a bilingual individual who, through a re-translation into the original language, confirms the comparability between the two versions [41].
- *Depression and Anxiety Stress Scale* (DASS-21) [42]; Italian version by Bottesi et al. 2015 [43]. To assess distress levels, a 21-item questionnaire on a 4-point Likert scale consisting of three dimensions (depression, anxiety, and stress) was used. Normal level ranges are 0–9 for depression, 0–7 for anxiety and 0–14 for stress; mild level ranges are 10–13 for depression, 8–9 for anxiety and 15–18 for stress; moderate level ranges are 14–20 for depression, 10–14 for anxiety and 19–25 for stress; severe level ranges are 21–27 for depression, 15–19 for anxiety and 26–33 for stress; extremely severe level ranges are 28 or more for depression, 20 or more for anxiety and 34 or more for stress. The Italian version of the DASS-21 showed good internal consistency (α = 0.90) and test–retest reliability over 2 weeks (r = 0.74) [43].
- *Psychological Well-Being Scales*, short form (PWBS) [44]; Italian version by Ruini et al. 2003 [45]. A 42-item questionnaire on a 6-point Likert scale consisting of six dimensions (autonomy, environmental mastery, personal growth, positive relations with others, purpose in life, and self-acceptance) was used to assess psychological well-being. Scores range between 7 and 42, with mid-range values indicating an optimal healthy range. The Italian version of the PWB showed good internal consistency and test–retest reliability for all six subscales, especially positive relations with others (r = 0.81), purpose in life (r = 0.81), and self-acceptance (r = 0.82) [45].
- *Dutch Eating Behavior Questionnaire* (DEBQ) [46]; Italian version by Dakanalis et al. 2013 [47]. A 33-item questionnaire on a 5-point Likert scale consisting of three dimensions (restrained, emotional, and external eating) was used to assess dysfunctional eating styles. Scores range from 1 to 5, with higher scores indicating more dysfunctional eating styles. The Italian version of the DEBQ showed a high internal consistency (α = 0.96 for emotional eating, α = 0.93 for external eating, and α = 0.92 for restrained eating) and test–retest reliability over 4 weeks (r = 0.93 for emotional eating, r = 0.92 for external eating, and r = 0.94 for restrained eating) [47].

### 3.3. Data Analyses

Statistical analyses were conducted with the Statistical Package for Social Science (SPSS) 29.0. Descriptive statistics were run to analyze sociodemographic characteristics of the sample (i.e., age, gender, marital status) and mean scores obtained from the administered questionnaires. Pearson correlations were run to explore the associations between sustainable eating behaviors and intention and DASS, PWB, and DEBQ scores.

A hierarchical linear regression analysis using the enter method was conducted to compare TPB and TBC predictors of the intention. Regarding the normality assumption, visual inspections were performed for all continuous variables used in parametric tests. Formal tests of normality, such as the Shapiro–Wilk test, were also conducted. While some variables exhibited statistically significant deviations from normality (*p* < 0.05), graphical inspections indicated that these deviations were not severe or extreme. Given the substantial sample size of this study, and following the central limit theorem, parametric test robustness to moderate normality violations was assumed [48]. Before conducting the regression analysis, the assumptions of linearity, independence of errors, homoscedasticity, and multicollinearity were checked. Linearity and homoscedasticity were verified through visual inspection of residual plots. The Durbin–Watson statistic was used to assess the independence of errors, showing an acceptable value of 2.03. All variance inflation factors (VIF) were below 2, indicating no significant multicollinearity. One outlier was detected based on standardized residuals. However, based on the assessed Cook’s distance value (<1), the data point did not impact the overall regression model; thus, it was not deleted for data analyses. After controlling for regression assumptions, the intention to engage in more sustainable eating behaviors was entered as the dependent variable (DV). Predictors of the intention proposed by the TPB model (attitude, social norms, and PBC) were instead entered as independent variables (IV) in block 1, and predictors of the intention proposed by the TBC model (attitude plus, affect, felt obligation, and habit) were entered as IV in block 2.

The SPSS process macro extension [49] was used for moderation analyses. Separate moderation analyses were conducted, including SHDB scores as DV, the intention to engage in more sustainable eating behaviors as IV, and DASS-21, PWB, and DEBQ scores as possible moderators of the relation between intention and behavior. The significance level for all statistical analyses was set to *p* ≤ 0.5.

## 4. Results

### 4.1. Sample Characteristics

Participants (N = 223) had a mean age of 29.49 ± 9.30 years and were mainly females (68.6%). The majority of them had obtained a master’s degree (45.7%), were employed at the moment of the assessment (74.4%), and were in a relationship (60.9%). Most of the participants declared themselves to be omnivorous (81.6%). Please see Table 1 for further details on the sociodemographic characteristics of the sample.

Regarding mean scores on the administered questionnaires, participants were in a healthy range for PWB and DEBQ, whereas they showed mild to moderate levels of DASS-21 depression, anxiety, and stress. They showed good scores on all SHDB dimensions, with the highest scores observed on SHDB-food consumption, cooking, and preservation. The overall levels of sustainable eating behaviors (SHDB-tot) were also good. Please see Table 2 for further details.

### 4.2. Bi-Directional Associations Between Sustainable Eating Behaviors and Intention, Psychological Well-Being, Dysfunctional Eating Styles, and Distress

Pearson correlational analyses showed significant moderate associations between intention to engage in sustainable eating behaviors and all SHDB dimensions (*p* < 0.001), except for food consumption, with the strongest correlation being with SHDB-food choices (r = 0.42). See Table S1.

Significant positive low correlations were found between SHDB-food choices and PWB-environmental mastery (r = 0.17, *p* = 0.01), personal growth (r = 0.14, *p* = 0.04) and positive relations with others (r = 0.14, *p* = 0.03); SHDB-food preservation and PWB-environmental mastery (r = 0.15, *p* = 0.02), personal growth (r = 0.19, *p* = 0.004), and autonomy (r = 0.15, *p* = 0.02); SHDB-cooking and PWB- environmental mastery (r = 0.16, *p* = 0.01), personal growth (r = 0.31, *p* < 0.001), positive relations with others (r = 0.13, *p* = 0.04), and autonomy (r = 0.19, *p* = 0.004); SHBD-food consumption and PWB-personal growth (r = 0.29, *p* < 0.001) and positive relations with others (r = 0.17, *p* = 0.01); SHDB-food disposal and PWB-personal growth (r = 0.14, *p* = 0.03); SHDB-total score and PWB-environmental mastery (r = 0.18, *p* = 0.008), personal growth (r = 0.24, *p* < 0.001), and positive relations with others (r = 0.17, *p* < 0.001). See Table S1.

Significant correlations between DASS scores and SHDB dimensions did not emerge. See Table S2.

Regarding dysfunctional eating styles, significant positive moderate correlations emerged between SHDB-food choices and DEBQ-restriction (r = 0.32, *p* < 0.001) and low between SHDB-total score and DEBQ-restriction (r = 0.22, *p* = 0.001). Please see Table S3 for further details.

### 4.3. Comparison of TPB and TBC Predictors of the Intention to Engage in Healthy and Sustainable Eating Behaviors

The first hierarchical regression analysis comparing TPB and TBC predictors of intention to engage in sustainable eating behaviors showed that Model 1 (TPB) accounted for 18% of the variance in intention with a medium effect size (f^2^ = 0.22). In comparison, Model 2 (TBC) accounted for 45% of the variance in intention with a large effect size (f^2^ = 0.83), with a significant increase in the predictive power of the model ($R^{2}\;$ = 0.45, $\Delta R^{2}$ = 0.28, F(4,215) = 27.27, *p* < 0.001). See Table 3 for the summary of regression models.

In Model 1, all three predictors (attitudes, subjective norms, and PBC) significantly predicted the intention to engage in sustainable eating behaviors. After adding TBC predictors of intention to the model, intention was significantly predicted by affect (β = 0.48, t = 8.13, *p* < 0.001) and felt obligation (β = 0.23, t = 4.06, *p* < 0.001), followed by social norms (β = 0.14, t = 2.13, *p* = 0.03). See Table 4 for the coefficients table.

### 4.4. Psychological Moderators in the Relation Between Intention and Behavior

**Figure 2:**
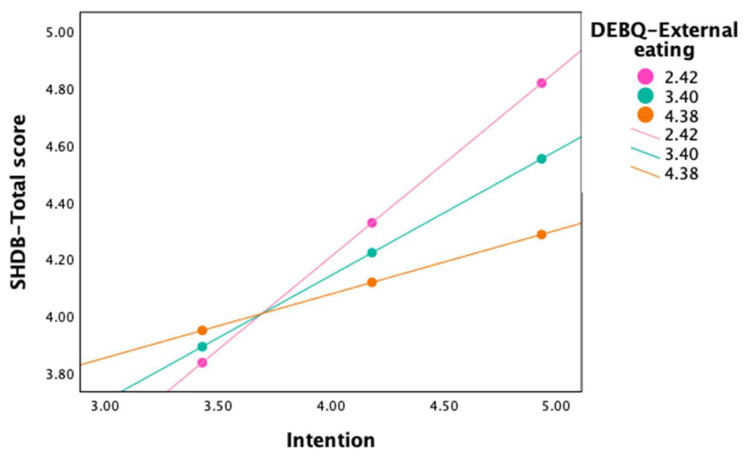
Interaction plot illustrating the moderating effect of DEBQ-external eating in the relation between intention and SHDB-total score. The three lines represent low (2.42), medium (3.40), and high (4.38) levels of DEBQ-external eating, corresponding to one standard deviation below the mean, the mean, and one standard deviation above the mean, respectively. Notes. DEBQ, Dutch Eating Behavior Questionnaire; SHDB, Sustainable and Healthy Dietary Behaviors.

DEBQ-external eating moderated the relation between intention and SHDB-total score. The overall regression model was statistically significant, with a medium to large effect size ($R^{2}$ = 0.22, F(3,219) = 21.12, *p* < 0.001, f^2^ = 0.28), as well as the interaction between intention and external eating (*p* < 0.001). As shown in Figure 2, the relationship between intention and behavior was stronger for individuals with lower levels of external eating. See Table 5 for the conditional effects of intention according to the three levels of external eating.

**Figure 3:**
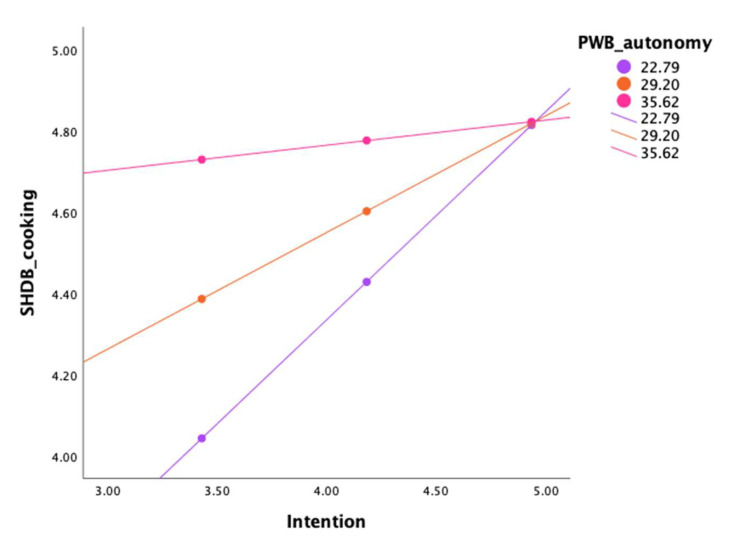
Interaction plot illustrating the moderating effect of PWB-autonomy in the relation between intention and SHDB-cooking. The three lines represent low (22.79), medium (29.20), and high (35.62) levels of PWB-autonomy, corresponding to one standard deviation below the mean, the mean, and one standard deviation above the mean, respectively. Notes. PWB, psychological well-being; SHDB, Sustainable and Healthy Dietary Behaviors.

PWB-autonomy moderated the relation between intention and SHDB-cooking. The overall regression model was statistically significant, with a low effect size ($R^{2}$ = 0.07, F(219,3) = 5.51, *p* = 0.001, f^2^ = 0.07), as well as the interaction between intention and PWB-autonomy (*p* = 0.04). As shown in Figure 3, the relationship between intention and behavior was stronger for individuals lower in autonomy. See Table 6 for conditional effects of intention according to the three levels of autonomy.

None of the other assessed psychological variables were found to moderate the relation between intentions and behaviors.

## 5. Discussion

The present cross-sectional study aimed to compare TPB and TBC predictors of intention to engage in more sustainable eating behaviors and to test the potential role of untested psychological moderators in the relation between intention and behavior.

Regarding the first aim, our hypothesis was confirmed. While the TPB accounted for a good amount of variance in intention with all its predictors reaching statistical significance and thereby supporting its predictive adequacy, the TBC model provided a better fit in predicting the intention to engage in more sustainable eating behaviors. Consistent with prior research comparing TBC and TPB models applied to healthy eating [12], plant-based dietary behaviors [13], and climate-friendly food choices [17], intention was significantly predicted by social norms, felt obligation, and affect.

Affect, representing the emotional value attributed to the behavior (e.g., “I would be happy to engage in healthier and more sustainable eating behaviors”), emerged as the strongest predictor of intention. This finding aligns with previous studies highlighting the significant role of anticipated affect in shaping behavioral intentions across various health-related behaviors [50,51,52,53]. Following this evidence, it has been suggested that, when planning future actions, individuals primarily consider the anticipated emotional state (i.e., well-being and happiness) associated with such actions [54]. In addition, our findings are also in line with recent evidence showing that intrinsic motives (i.e., being healthy, feeling better) represent one of the most significant predictors of healthy eating [55].

Feelings of obligation (e.g., “I feel obligated to change my eating behaviors towards more sustainable ones”) and social norms (e.g., “I will change my behavior because significant others would criticize me if I do not do it”) also significantly predicted intention. The literature widely supports the role of social influences in eating behaviors, such as adapting one’s own food choices to those of others and using eating behaviors to convey a positive impression on others [56]. In line with our results, prior research on pro-environmental behaviors has also highlighted the role of external factors, such as social pressure, in determining behavioral change [57,58,59].

The prominent role of affect, felt obligation, and social norms highlighted in our study through the direct comparison of theoretical psychological models, confirms that behavioral intention to adopt sustainable eating behaviors is driven both by internal motivations (i.e., affect) and external pressures (i.e., feeling obligated), also in line with previous studies on sustainable food consumption [6,60].

Concerning the second aim of this study, our hypothesis was partially confirmed. Dysfunctional eating styles and PWB, but not distress levels, were identified as moderators in the relation between intention and behavior.

External eating significantly moderated the relation between intention and behavior regarding overall sustainable eating behaviors. Specifically, the association between intention and behavior was stronger in individuals with lower levels of external eating, the tendency to eat in response to external stimuli regardless of the internal state of hunger or satiety. It has been widely supported that individuals with external eating styles tend to prefer unhealthy and palatable foods rather than healthy options [61] and often show low self-control and increased impulsiveness [62,63,64]. Self-control is the attempt to modify one’s behaviors, emotions, and thoughts to reach long-term goals [65]. Individuals with higher self-control typically employ more effective self-regulation strategies than those with lower self-control [66,67], who instead are more prone to impulsive actions [68]. Our findings on the moderating role of low external eating may reflect the application of higher self-control strategies. This also suggests a tendency to better inhibit immediate, short-term rewards (i.e., eating unhealthy food in response to external stimuli) in favor of long-term outcomes (i.e., adhering to healthy and sustainable diets). This result aligns with prior research supporting the association between self-control and healthy and sustainable eating behaviors [69,70]. Specifically, it has been suggested that sustainability practices stem from the ability to regulate cognition and behaviors [69], further corroborating our finding on the importance of regulating eating behaviors.

Regarding PWB, autonomy significantly moderated the relation between intention and sustainable eating in terms of food cooking (i.e., cooking skills that allow cooking in a way that minimizes food waste and the use of non-sustainable tools, such as plastic cutlery). The association between intention and behavior was stronger among individuals with lower levels of autonomy (i.e., individuals who are concerned about the evaluations of others, rely on the judgment of others to make important decisions, and conform to social pressure) [71]. While this finding might initially seem to be in contrast with the earlier result on external eating, given that low autonomy is characterized by lower self-control [72], it is noteworthy that autonomy in our study moderated the relationship between intention and cooking skills specifically, rather than overall sustainable eating behaviors. Although we did not collect information on the cooking habits of our sample (i.e., whether they cooked their food or someone else cooked for them), this finding may reflect a lower tendency among our participants to cook for themselves and to be less autonomous when it comes to culinary choices. In this context, it is understandable that their behavioral shift toward more sustainable cooking practices could be influenced by their limited autonomy and may therefore depend on the actions of others. Our result on the moderating role of low autonomy also aligns with the role of feelings of obligation and social norms in predicting intention, suggesting that for some individuals, the shift towards more sustainable eating behaviors may be influenced by social obligations rather than intrinsic motivation. Lower levels of autonomy, where choices are not solely driven by self-determined reasons that might conflict with policymakers’ recommendations, may actually promote more sustainable eating behaviors. However, this finding warrants clinical attention, as both exceedingly low and high levels of autonomy may be counterproductive in some instances. On the one hand, extrinsic motivation stemming from low autonomy may lead to temporary behavioral changes that do not persist over time [73]. Conversely, excessively high levels of autonomy may contribute to poor eating-related outcomes (i.e., weight loss) [31]. Individuals with high levels of autonomy might be over-confident about their skills and thus believe they cannot learn from others’ advice [31]. Therefore, maintaining balanced psychological well-being and avoiding both extremes appear essential for fostering lasting behavioral change.

The potential influence of PWB and dysfunctional eating styles on sustainable eating behaviors was further supported by correlational analyses. Significant correlations, although low, emerged between sustainable eating behaviors and PWB, with personal growth standing out as particularly relevant. High levels of personal growth—characterized by a sense of ongoing development, openness to new experiences, and the ability to see self-improvement over time [71]—were strongly associated with greater sustainable eating behaviors. Conversely, individuals with lower levels of personal growth, who struggle to adopt new attitudes or behaviors, may find behavioral change more challenging. This finding aligns with prior research showing that openness to experiences is related to pro-environmental behaviors [74]. Additionally, personal growth may foster self-efficacy and agency, essential factors for developing and maintaining new behaviors [75], and enhance a sense of fulfillment and purpose related to pro-environmental behaviors [76]. In our sample, higher levels of PWB–environmental mastery, defined as the ability to manage the environment, make effective use of surrounding opportunities, and create contexts suitable to personal needs and values [71], were also positively associated with greater sustainable eating behaviors. Thus, positive psychological functioning may play an important role in driving behavioral changes towards more sustainable eating behaviors, in line with prior research revealing a link between pro-environmental, sustainable, and ecological behaviors and positive psychological factors (i.e., happiness and psychological well-being) [76,77].

Notably, there were also moderate, positive correlations between sustainable food choices and DEBQ-restrained eating, defined as the conscious restriction of food intake to manage body weight. Increased food restriction was associated with greater sustainable eating behaviors in our sample. This finding needs to be further explored in future studies, considering the well-known link between excessive attention to healthy eating and the onset of disordered-eating symptoms, such as orthorexia nervosa [78,79,80].

### Limitations, Future Directions, and Clinical Implications

The present findings should be interpreted in light of some limitations. Firstly, while our sample size met the requirements set by prior power analyses, the sample is not representative of the general population, and thus, results cannot be generalized. Our sample was skewed toward a higher proportion of female participants and individuals with higher levels of education, factors that may limit the generalizability of our findings. Secondly, while we checked for key statistical assumptions for our parametric analyses, some continuous variables exhibited statistically significant deviations from normality. Although graphical inspections revealed that these deviations were not extreme, and the large sample size supports the robustness of our parametric tests, it is important to acknowledge that ideal normality was not universally met. Furthermore, the assessment of our variables relied on self-report questionnaires, which are known to be subject to several biases, such as social desirability or recall bias. In addition, sustainable eating behaviors relied on a questionnaire (i.e., SHDBs) that has yet to be psychometrically validated in the Italian population. Moreover, although a backward translation method was employed for the Italian version, the second review was conducted by a single expert, and cognitive debriefing or field testing was not performed. These aspects may represent limitations in fully ensuring the optimal quality of the translation. Another important limitation concerns the low correlations between sustainable eating behaviors and the assessed psychological variables, particularly psychological well-being and distress. The relation between these variables may be more complex than expected and could be mediated by additional variables not considered in the present study. In addition, the current study included participants from the general population, who might be characterized by substantial individual variability. Studies conducted in more targeted populations, such as individuals highly attentive to sustainable diets or experiencing elevated levels of distress, may reveal stronger associations between the variables mentioned above. Furthermore, due to the study’s cross-sectional design, we cannot examine the longitudinal trends in sustainable eating behaviors and associated psychological variables.

Future studies should address these limitations by including larger and more heterogeneous samples in terms of sociodemographic characteristics. Studies with stronger statistical power may be helpful in exploring further the role of certain psychological variables that did not emerge as relevant in the present study, such as psychological distress. Potential mediators in the relation between sustainable eating and psychological functioning in well-being and distress should also be tested. Moreover, although investigating sustainable eating in the general population is essential for informing policymakers on strategies to promote healthier and more sustainable eating behaviors, additional research in clinical populations is needed. Including more specific populations could clarify the relation between sustainable eating behaviors and psychological functioning. In addition, our findings revealed a significant association between restrained and sustainable eating behaviors, raising potential concerns for clinicians. Future studies could explore whether the clinical profile of orthorexia nervosa, typically characterized by an excessive focus on healthy eating, may be extended to sustainable eating behaviors as well [81]. Longitudinal and intervention studies are also needed to test possible intervention strategies to encourage more sustainable food choices in healthy and clinical populations.

Despite these limitations, this study provides valuable insights into the role of specific psychological factors influencing sustainable eating behaviors. To our knowledge, this is the first study applying the TBC, a recently developed theoretical model, to the context of sustainable eating intentions and behaviors. In the current study, the TBC has been further extended with additional clinical psychological variables that have not been previously tested within this specific context. A novel, integrative predictive model was developed and has been found to predict sustainable eating intentions effectively. Specifically, affect, social norms, and felt obligation emerged as significant predictors of intention, while the other additional TBC predictors of intention (attitudes plus and habit) did not significantly contribute to its variance. Importantly, before incorporating the full TBC, all TPB predictors (social norms, attitudes, and PBC) were also found to significantly predict intentions. In light of these findings, future studies could further test predictive models for sustainable eating intentions by focusing on the significant predictors identified in the present study. This approach could lead to the development of shorter, easier-to-use, and methodologically sound theoretical models. Additionally, clinical psychological variables, including psychological well-being and dysfunctional eating styles, have been found to play an important role in implementing sustainable and healthy eating behaviors. These findings offer several important practical implications. Firstly, internal and external motivators, such as emotional ones and socially based ones, should be considered and incorporated in policymaking to foster sustainable eating behaviors in the general population. Policymakers should also consider that individual differences in psychological well-being levels—autonomy in particular—may affect the adherence to sustainable eating guidelines. In addition, interventions promoting such behaviors should consider the bidirectional relations between eating-related sustainable behaviors and individuals’ psychological functioning. Enhancing individuals’ positive psychological functioning and promoting more functional eating styles may foster more conscious and sustainable eating behaviors. In turn, being more sustainable in terms of food choices and overall eating and cooking-related behaviors may improve one’s psychological well-being and relationship with food, potentially preventing mental health issues. Due to the intricate relationship between psychological factors and sustainable eating behaviors, a collaboration between healthcare professionals and policymakers is needed to develop adequate and effective interventions to promote more sustainable eating habits in the general population.

## 6. Conclusions

This study advances our understanding of the psychological mechanisms underlying sustainable eating behaviors in healthy individuals by comparing two theoretical models—TPB and TBC—and exploring the moderating role of psychological well-being and dysfunctional eating styles in the relation between intention and behavior. The TBC emerged as a more comprehensive framework, highlighting the pivotal role of affect, felt obligation, and social norms in predicting behavioral intentions. Furthermore, individual psychological characteristics such as external eating and autonomy moderated the intention–behavior link, underscoring the complexity of translating sustainable intentions into actions. Specifically, in participants with lower levels of external eating—the tendency to eat in response to external stimuli rather than physical hunger—the intention-behavior link was stronger, underscoring the role of self-regulation as a potential psychological mechanism underlying behavioral change towards more sustainable eating. Similarly, this link was stronger also in participants with lower levels of autonomy—those who are concerned about the evaluations of others, rely on the judgment of others to make important decisions, and conform to social pressure, suggesting that for some individuals behavioral change may be more strongly driven by others’ actions and expectations. Altogether, our findings suggest that both internal motivators (e.g., emotional value) and external pressures (e.g., social expectations) influence sustainable eating intentions, while both internal (e.g., self-regulation) and external (e.g., autonomy) psychological mechanisms may shape actual behavior. Importantly, the study emphasizes the need for integrative intervention strategies that not only promote sustainable food choices but also enhance individuals’ psychological well-being and self-regulatory capacities. Future research should expand on these results by testing tailored interventions in diverse and clinical populations, aiming to foster long-term behavioral change. 

## Figures and Tables

**Table 1:** Sociodemographic characteristics of the sample.

Variables	M (SD)
**Age**	29.49 (9.30)
	**N (%)**
**Gender**	
Female	153 (68.6%)
Male	69 (30.9%)
Non-binary	1 (0.4%)
**Education**	
No qualification	1 (0.4%)
Middle school	3 (1.3%)
High school	59 (26.5%)
Bachelor degree	44 (19.7%)
Master degree	102 (45.7%)
Other	14 (6.3%)
**Occupation**	
Employee	166 (74.4%)
Student/internee	30 (13.4%)
Unemployed	8 (3.6%)
Retired	1 (0.4%)
Other	18 (8.1%)
**Marital status**	
Single	66 (29.6%)
In a relationship	136 (60.9%)
Married	16 (7.2%)
Separated/divorced	5 (2.2%)
**Nutrition**	
Omnivorous	182 (81.6%)
Lacto-vegetarian	5 (2.2%)
Ovo-vegetarian	1 (0.4%)
Lacto–ovo vegetarian	9 (4.0%)
Vegan	4 (1.8%)
Flexitarian	15 (6.7%)
Pesco–lacto–ovo vegetarian	7 (3.1%)

Notes. M, mean; SD, standard deviation.

**Table 2:** Mean scores on PWB, DASS-21, DEBQ, and SHBD total score.

Variables	M (SD)
**Sustainable and Healthy Dietary Behaviors (SHDBs)**	
Food choices	3.68 (0.98)
Food preservation	4.59 (1.2)
Cooking	4.6 (0.95)
Food consumption	5.3 (0.91)
Food disposal	4.52 (0.88)
Total	4.20 (0.75)
**Depression and Anxiety Stress Scale (DASS)**	
Depression	13.63 (10.69)
Anxiety	10.88 (9.66)
Stress	19.83 (10.33)
**Psychological Well-Being Scale (PWBS)**	
Autonomy	29.20 (6.41)
Environmental mastery	27.35 (4.71)
Personal growth	33.70 (4.92)
Positive relations with others	31.97 (5.62)
Purpose in life	29.89 (5.70)
Self-acceptance	28.74 (6.96)
**Dutch Eating Behavior Questionnaire (DEBQ)**	
Restriction	2.54 (1.05)
External eating	3.40 (0.97)
Emotional eating	2.42 (1.15)

Notes. M, mean; SD, standard deviation.

**Table 3:** Summary of regression models.

	$R^{2}$	${\Delta R}^{2}$	F (*df*)	ΔF	p(ΔF)	f^2^
**Model 1 (TPB)**	0.178	-	17.03 (3, 219)	-	-	0.22
**Model 2 (TBC)**	0.453	0.281	28.52 (4, 215)	28.52	<0.01	0.83

Notes. *df*, degrees of freedom; TBC, theory of behavioral choice; TPB, theory of planned behavior. Model 1 (TPB) includes attitudes, subjective norms, and PBC. Model 2 (TBC) includes attitudes, subjective norms, PBC, attitude plus, habits, affect, and felt obligation. Outcome variable: intention.

**Table 4:** Coefficients table.

	B	SE	β	t (*p*)	95% CI
**Model 1 (TPB)**					
**Attitudes**	0.13	0.06	0.15	2.11 **(0.04)**	[0.01, 0.25]
**Subjective norms**	0.14	0.04	0.28	3.70 **(<0.01)**	[0.06, 0.22]
**Perceived behavioral control (PBC)**	0.17	0.04	0.30	4.29 **(<0.01)**	[0.09, 0.25]
**Model 2 (TBC)**					
**Attitudes**	0.07	0.06	0.08	1.22 (0.22)	-
**Social norms**	0.07	0.03	0.14	2.13 **(0.03)**	[0.01, 0.13]
**PBC**	0.05	0.04	0.09	1.38 (0.17)	-
**Attitude plus**	0.05	0.05	0.08	1.14 (0.25)	-
**Habits**	0.005	0.03	0.01	0.18 (0.86)	-
**Felt obligation**	0.14	0.03	0.23	4.06 **(<0.01)**	[0.08, 0.20]
**Affect**	0.50	0.06	0.48	8.13 **(<0.01)**	[0.38, 0.62]

Notes. TBC, theory of behavioral choice; TPB, theory of planned behavior. Significant values in bold.

**Table 5:** Conditional effects of intention on behavior according to different levels of external eating.

DEBQ-External Eating	Effect	SE	t	*p*	95% CI
2.42	0.65	0.09	7.49	**<0.01**	[0.48, 0.82]
3.40	0.44	0.06	7.25	**<0.01**	[0.31, 0.56]
4.38	0.22	0.08	2.78	**<0.01**	[0.06, 0.38]

Notes. DEBQ, Dutch Eating Behavior Questionnaire; SE, standard error. Significant values in bold.

**Table 6:** Conditional effects of intention on behavior according to different levels of autonomy.

PWB-Autonomy	Effect	SE	t	*p*	95% CI
22.79	0.51	0.15	3.30	**<0.01**	[0.21, 0.82]
29.20	0.29	0.10	2.73	**<0.01**	[0.08, 0.49]
35.62	0.06	0.15	0.42	0.68	-

Notes. PWB, psychological well-being; SE, standard error. Significant values in bold.

## Data Availability

Data is available upon reasonable request from the corresponding author.

## Supplementary Materials

The following supporting information can be downloaded at: https://www.mdpi.com/article/10.3390/nu17152391/s1, Table S1: Pearson’s r correlations between intention, SHDB, and PWB; Table S2: Pearson’s r correlations between intention, SHDB, and DASS; Table S3: Pearson’s r correlations between intention, SHDB, and DEBQ.
