# Supplementary material for: Psychological Well-Being and Dysfunctional Eating Styles as Key Moderators of Sustainable Eating Behaviors: Mind the Gap Between Intention and Action
Source: Nutrients. 2025 Jul 22;17(15):2391. doi: 10.3390/nu17152391 (PMC12348166; doi:10.3390/nu17152391)
Supplement: Supplementary file 1 [file nutrients-17-02391-s001.zip › nutrients-3748267-supplementary.pdf]

supplementary materials

Table S1. Pearson's r correlations between intention, SHDB and PWB.

	Intention	SHDB-food choices	SHDB- food preservation	SHDB- cooking	SHDB- food consumption	SHDB- food disposal	SHDB-total	PWB-environmental mastery	PWB- personal growth	PWB- positive relation with others	PWB-autonomy	PWB-purpose in life	PWB- self-acceptance
**Intention**	1	.417**	.182**	.259**	.115	.247**	.405**	.194**	-.009	.038	.069	-.070	.090
**SHDB-food choices**	.417**	1	.352**	.509**	.338**	.353**	.887**	.168*	.137*	.143*	.020	.014	.072
**SHDB- food preservation**	.182**	.352**	1	.591**	.339**	.364**	.621**	.149*	.191**	.097	.151*	.105	.072
**SHDB-cooking**	.259**	.509**	.591**	1	.449**	.492**	.787**	.163*	.314**	.135*	.192**	.077	.080
**SHDB-food consumption**	.115	.338**	.339**	.449**	1	.414**	.534**	.079	.296**	.169*	.090	.133*	.173**
**SHDB-food disposal**	.247**	.353**	.364**	.492**	.414**	1	.624**	.017	.143*	.131	.039	.034	.045
**SHDB-total**	.405**	.887**	.621**	.787**	.534**	.624**	1	.177**	.245**	.175**	.100	.062	.098
**PWB-autonomy**	.069	.020	.151*	.192**	.090	.039	.100	.413**	.391**	.318**	1	.393**	.488**
**PWB-environmental mastery**	.194**	.168*	.149*	.163*	.079	.017	.177**	1	.403**	.456**	.413**	.542**	.694**
**PWB-personal growth**	-.009	.137*	.191**	.314**	.296**	.143*	.245**	.403**	1	.514**	.391**	.536**	.489**
**PWB-positive relations with others**	.038	.143*	.097	.135*	.169*	.131	.175**	.456**	.514**	1	.318**	.474**	.587**
**PWB-purpose in life**	-.070	.014	.105	.077	.133*	.034	.062	.542**	.536**	.474**	.393**	1	.639**
**PWB-self-acceptance**	.090	.072	.072	.080	.173**	.045	.098	.694**	.489**	.587**	.488**	.639**	1

**p<0.01, *p<0.05.

Table *S*2. Pearson's r correlations between intention, SHDB and DASS.

	Intention	SHDB-food choices	SHDB-food preservation	SHDB-cooking	SHDB-food consumption	SHDB-food disposal	SHDB-total	DASS-depression	DASS-anxiety	DASS-stress
**Intention**	1	.417**	.182**	.259**	.115	.247**	.405**	.064	.092	.065
**SHDB-food choices**	.417**	1	.352**	.509**	.338**	.353**	.887**	.030	.067	.098
**SHDB-food preservation**	.182**	.352**	1	.591**	.339**	.364**	.621**	-.061	-.033	.014
**SHDB-cooking**	.259**	.509**	.591**	1	.449**	.492**	.787**	-.020	-.086	.063

SHDB-food consumption	.115	.338**	.339**	.449**	1	.414**	.534**	-.070	-.106	-.027
SHDB-food disposal	.247**	.353**	.364**	.492**	.414**	1	.624**	.061	.037	.082
SHDB-total	.405**	.887**	.621**	.787**	.534**	.624**	1	.009	.012	.092
DASS-depression	.064	.030	-.061	-.020	-.070	.061	.009	1	.688**	.706**
DASS-anxiety	.092	.067	-.033	-.086	-.106	.037	.012	.688**	1	.671**
DASS-stress	.065	.098	.014	.063	-.027	.082	.092	.706**	.671**	1

**p<0.01.

Table S3. Pearson's r correlations between intention, SHDB and DEBQ.

	Intention	SHDB-food choices	SHDB-food preservation	SHDB-cooking	SHDB-food consumption	SHDB-disposal	SHDB-total	DEBQ-restrained eating	DEBQ-external eating	DEBQ-emotional eating
Intention	1	.417**	.182**	.259**	.115	.247**	.405**	.244**	.126	.016
SHDB-food choices	.417**	1	.352**	.509**	.338**	.353**	.887**	.321**	-.086	.046
SHDB-food preservation	.182**	.352**	1	.591**	.339**	.364**	.621**	.113	-.135*	.003
SHDB-cooking	.259**	.509**	.591**	1	.449**	.492**	.787**	.047	-.046	-.031
SHDB-food consumption	.115	.338**	.339**	.449**	1	.414**	.534**	-.131	.071	-.036
SHDB-food disposal	.247**	.353**	.364**	.492**	.414**	1	.624**	.047	.105	-.043
SHDB-total	.405**	.887**	.621**	.787**	.534**	.624**	1	.223**	-.059	.009
DEBQ-restrained eating	.244**	.321**	.113	.047	-.131	.047	.223**	1	.047	.224**
DEBQ-external eating	.126	-.086	-.135*	-.046	.071	.105	-.059	.047	1	.257**
DEBQ-emotional eating	.016	.046	.003	-.031	-.036	-.043	.009	.224**	.257**	1

**p<0.01, *p<0.05.
